# Sit‐to‐Stand Power From 2D Pose Estimation as an Indicator of Muscle Strength in Older Adults

**DOI:** 10.1002/jcsm.70208

**Published:** 2026-01-26

**Authors:** Wonjae Hwang, Dain Shim, Joowan Kim, Kyung Rok Oh, Sun Gun Chung, Jaewon Beom, Myung Woo Park, Kyung Su Kim, Joonghee Kim, Chul Hyun Park, Keewon Kim

**Affiliations:** ^1^ Department of Rehabilitation Medicine Seoul National University Hospital Seoul South Korea; ^2^ The Armed Forces Daejeon Hospital Daejeon South Korea; ^3^ Department of Rehabilitation Medicine Seoul National University College of Medicine Seoul South Korea; ^4^ Institute of Aging, Medical Research Center Seoul National University Seoul South Korea; ^5^ Department of Rehabilitation Medicine, Seoul National University Bundang Hospital Seoul National University College of Medicine Seongnam South Korea; ^6^ Department of Rehabilitation Medicine Seoul National University College of Medicine, Seoul Metropolitan Government‐Seoul National University Boramae Medical Center Seoul South Korea; ^7^ Department of Emergency Medicine Seoul National University Hospital Seoul South Korea; ^8^ Department of Emergency Medicine Seoul National University Bundang Hospital Seongnam South Korea; ^9^ Department of Physical and Rehabilitation Medicine, Kangbuk Samsung Hospital Sungkyunkwan University School of Medicine Seoul South Korea

**Keywords:** muscle power, older people, pose estimation, sarcopenia, sit to stand

## Abstract

**Background:**

The sit‐to‐stand (STS) test is a crucial tool for sarcopenia assessment. However, the two most widely used diagnostic frameworks differ in their conceptualization of the test. The European Working Group on Sarcopenia in Older People 2 views the STS test primarily as a proxy for muscle strength, while the Asian Working Group for Sarcopenia 2019 (AWGS 2019) classifies it as a measure of physical performance. This discrepancy poses challenges to conceptual clarity in both research and practice. Recent advancements in pose‐estimation algorithms allow for kinematic assessment using a standard handheld devices, providing a simple and cost‐effective alternative to conventional motion‐analysis.

**Methods:**

A total of 129 community‐dwelling older adults (mean age 71.8 years) who could ambulate independently underwent assessments of muscle strength (knee extensor and handgrip strength), physical performance (usual walking speed, Timed Up and Go [TUG] velocity and Short Physical Performance Battery [SPPB]) and muscle mass (appendicular skeletal muscle index by bioelectrical impedance analysis). Kinematic analysis of the 30‐s sit‐to‐stand (30‐s STS) was conducted using 2D pose estimation to derive peak STS power and joint angles. For validation, a subset of 20 participants completed 30‐s STS trials that were recorded concurrently using 2D pose estimation, optical motion capture and force‐plate recordings. The correlation between peak STS power, five times sit‐to‐stand (5x‐STS) time, and 30‐s STS repetitions with clinical indicators was examined.

**Results:**

Peak STS power correlated strongly with muscle strength and mass (knee extensor strength *r* = 0.64; handgrip *r* = 0.64; ASMI *r* = 0.70; all *p* < 0.001) but only weakly with physical performance (usual walking speed: *r* = 0.19, *p* = 0.030; TUG velocity and SPPB: *p* > 0.05). In contrast, the 5x‐STS time and 30‐s STS repetitions showed moderate to strong associations with physical performance (e.g., SPPB *r* = −0.79 and 0.52, respectively; both *p* < 0.001) but did not correlate with strength or muscle mass. Despite systematic biases between pose estimation and the reference, the agreement was high (intraclass correlation coefficient for peak STS power = 0.94; joint‐angles = 0.83–0.89). In sex‐stratified, within‐subject mixed‐effects models, changes in posture and timing explained modest variance in ΔPeak STS power (marginal *R* ^2^ = 0.024 in men; 0.138 in women).

**Conclusions:**

Peak STS power correlated more strongly with muscular strength, while 5x‐STS and 30‐s STS tests were more closely related to physical performance. These findings align better with and provide stronger support for the AWGS 2019 framework.

## 1 Introduction

Adults aged 65 years and older made up 10.3% of the global population in 2024, and this percentage is projected to rise to 20.7% by 2074 [1]. Against this demographic shift, sarcopenia—a progressive and generalized skeletal‐muscle disorder characterized by accelerated loss of muscle mass and function—has become a major public health concern. Its global prevalence is approximately 10% among older adults [2], and it is associated with increased risks of falls, functional decline, frailty and mortality [3].

For the diagnosis of sarcopenia, the two most widely used frameworks—the Asian Working Group for Sarcopenia 2019 (AWGS 2019) [4] and the European Working Group on Sarcopenia in Older People 2 (EWGSOP2) [5]—integrate three domains: muscle mass, muscle strength and physical performance. Muscle mass is typically assessed using dual‐energy X‐ray absorptiometry or bioelectrical impedance analysis; muscle strength is measured with handgrip dynamometry; and physical performance is evaluated through gait speed or the Short Physical Performance Battery (SPPB). Additionally, the sit‐to‐stand (STS) test is a key tool in this assessment process.

The STS test is widely used across diverse populations, including community‐dwelling older adults and individuals with various comorbidities [6]. However, its classification within sarcopenia frameworks is inconsistent. The AWGS 2019 classifies the STS test as a measure of physical performance [4, 7], while the EWGSOP2 considers it an assessment of muscle strength [5]. Conceptually, rising from a chair requires generating sufficient force to overcome body weight, suggesting a strength component. Nevertheless, multiple studies have reported only moderate correlations between STS performance and lower‐limb muscle strength [8], indicating that the STS test cannot be regarded as a pure measure of strength. Since standing up is a common mobility task in daily life, the STS test also aligns with physical performance, which is often defined as ‘an objectively measured whole‐body function related to mobility’ [9].

Recent advances in pose estimation algorithms enable biomechanical assessments using only a standard handheld device [10]. These algorithms employ computer vision techniques to track human movement and accurately estimate body key point coordinates without the need for markers. When applied to the sit‐to‐stand (STS) test, pose estimation can provide more refined STS metrics while minimizing the burden of data collection. In this work, we focus on STS power—the mechanical power generated during the STS transition—which has demonstrated validity, reliability and prognostic value for sarcopenia, frailty and disability [11, 12, 13]. Traditionally, quantifying STS power has required specialized instrumentation, such as force plates [14], linear position transducers [15] or three‐dimensional accelerometers [16]. A pose‐estimation–based workflow may offer a simpler, more convenient and lower‐cost alternative, utilizing only a smartphone or tablet.

Accordingly, we pursued two main objectives. First, we aimed to determine whether the STS test is better understood as an indicator of muscle strength or physical performance. To achieve this, we examined the correlations between STS metrics (30‐s STS and 5x‐STS) and pose‐estimation–derived peak STS power (peak P_PE_) with measures of physical performance, muscle mass and muscle strength. For preliminary validation, we compared peak P_PE_ with a reference peak power (peak P_ref_) obtained from simultaneous motion‐capture and force‐plate measurements, in a subgroup of 20 participants. Second, we evaluated whether kinematic factors during the STS test influence peak P_PE_, with the goal of informing future recommendations for test protocols.

## 2 Material and Methods

### 2.1 Study Design and Participants

This study was designed as a prospective, observational cross‐sectional study. Participants were recruited between July 2022 and August 2025 at Seoul National University Hospital, a tertiary referral hospital in Seoul, South Korea. Recruitment efforts included advertisements on posters displayed within the hospital and on public transportation, such as buses and subways. Eligible participants were individuals aged 65 years or older who could walk independently and had no significant neurological, musculoskeletal or medical conditions that could affect their daily living. All participants provided informed consent, and the study protocol received approval from the Institutional Review Board of Seoul National University Hospital (IRB No. 2104‐125‐1213).

All participants underwent assessments of anthropometry (body mass and height), muscle strength (knee extensor and handgrip strength) and physical performance (usual walking speed, Timed Up and Go [TUG] and the Short Physical Performance Battery [SPPB]). Of these, 109 completed a single 30‐s sit‐to‐stand (STS) trial, during which kinematics were quantified using pose estimation. The remaining 20 participants formed a validation subgroup and that underwent simultaneous video‐based pose estimation, optical motion capture and force‐plate recordings during the 30‐s STS. Comorbidities were evaluated by asking participants if they had ever been diagnosed with any chronic diseases by a physician. We specifically recorded the presence of diabetes, hypertension, dyslipidaemia, heart disease (such as coronary artery disease, heart failure or arrhythmia), a history of cancer, thyroid disease, chronic liver disease, chronic lung disease and chronic kidney disease.

### 2.2 Sample Size Calculation

The sample size for total participants was set to ensure sufficient power to detect small associations (*r* = 0.3) between kinematic outcomes and physical performance measures. With an alpha level of 0.05 and a power of 0.80 (two‐tailed), a minimum of 84 participants was required [17]. To achieve a more accurate estimation of the association, we recruited participants beyond the minimum required sample size, resulting in a total of 129 participants.

To calculate the sample size for the validation group, we first assessed the Pearson correlation coefficient between peak P_PE_ and peak P_ref_ in the first 10 participants. We based the sample size calculation on the lower bound of the 95% confidence interval for the observed correlation (i.e., *r* = 0.92) and aimed for a desired half‐width of 0.10. Consequently, the minimum required sample size was determined to be 16 [18]. An additional four participants were recruited beyond the minimum sample size, resulting in a total of 20 participants in the validation group.

### 2.3 30‐s Sit‐to‐Stand Test (30‐s STS)

**FIGURE 1:**
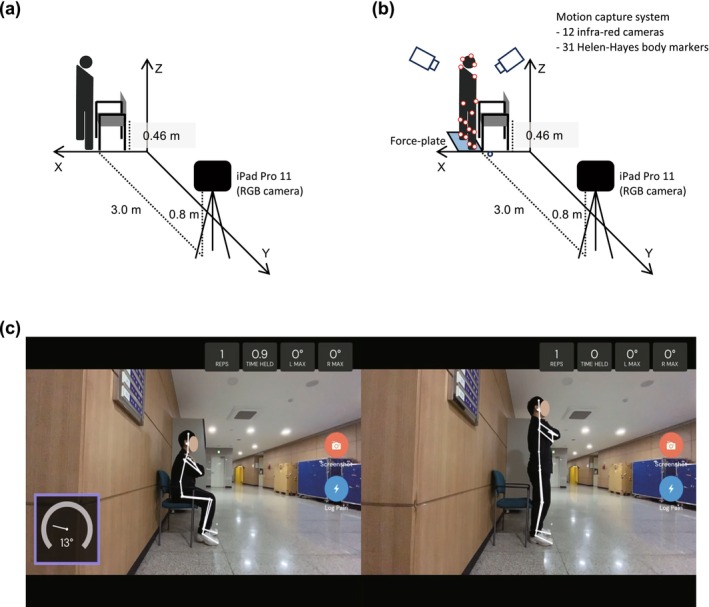
Experimental settings. (a) Set‐up for the 30‐s sit‐to‐stand (30‐s STS) test. An RGB camera (iPad Pro 11) is positioned 3 m from and perpendicular to the participant's sagittal plane, mounted on a tripod 0.8 m above the floor. (b) Validation subgroup (*n* = 20): concurrent recordings from a floor‐embedded force plate beneath the feet and an optical motion‐capture system. (c) Screenshot of the application showing the pose‐estimation model during the 30‐s STS test.

Participants were instructed to perform a 30‐s sit‐to‐stand‐to‐sit (30‐s STS) test while being recorded with the RGB camera of an Apple iPad Pro 11 (Apple Inc., Cupertino, CA, USA). The camera was positioned 3 m perpendicular to the subject's sagittal plane and mounted on a tripod at a height of 0.8 m above the ground (Figure 1a). The alignment was verified using a level to ensure horizontal accuracy. During the test, subjects began from a seated position in a chair and were instructed to complete as many repetitions of sit‐to‐stand‐to‐sit as possible within 30 s. They were required to fully extend their knees when standing and to sit down until their hips fully touched the chair. Additionally, subjects were instructed to cross their arms over their chest. A chair without armrests, measuring 46 cm in height, was used, and participants were allowed to wear comfortable footwear during the test. Throughout the 30‐s STS, a trained research nurse manually recorded the total number of repetitions, and pose‐estimation–derived kinematic data were extracted for subsequent analyses.

### 2.4 Pose‐Estimation–Derived Parameters

A customized pose estimation model (ViFive Inc., Boulder, Colorado) was utilized to extract time‐series data for key points from 2D images. The application's running screen is presented in Figure 1c. The model was adapted from the stacked hourglass architecture [19] and incorporates proprietary structural modifications to enhance both accuracy and processing speed. A tailored random forest classifier was integrated into the framework to optimize computational efficiency and precision. The model was trained on an extensive dataset that included a diverse range of age groups, body types and environmental conditions to promote generalizability. The 2D images were captured at a resolution of 800 × 600 pixels and at a rate of 30 frames per second. From each image, the following key points were extracted: the vertex, neck, shoulder, elbow, wrist, hip, knee and ankle. Analysis was conducted using either the right or left side, as captured in the sagittal image.

A fourth‐order Butterworth low‐pass filter with a cut‐off frequency of 4 Hz was applied to smooth the time series data for key points. Key points were corrected for perspective distortion using a pinhole‐camera model. It was assumed that, the hip, knee and ankle key points are consistently positioned 0.5 times the pelvic‐width closer to the camera than the body midline, while the shoulder, elbow, and wrist key points are positioned 0.5 times the shoulder‐width closer. The ratios of shoulder and pelvic width to participant height were obtained from Winter's anthropometric data [20]. (See Table S1.) Subsequently, for each frame, we computed the vertical vertex–ankle distance D along the z‐axis, as well as the hip‐ and knee‐flexion angles. Their sum defined θ, where a smaller θ indicates a more upright posture. We characterized the D–θ relationship by fitting a second‐order polynomial to the scatter using ordinary least squares. The peak segmental length, D_peak_, was taken as the maximum of the fitted quadratic. Based on Winter's anthropometry [20], we set D_peak_ = 0.961• h and used this to compute the pixel‐to‐distance conversion factor.

To compute the muscle power during 30‐s STS, we first estimated the centre‐of‐mass (CoM) position (*r*_CoM_) as a weighted average of body key points (*r*_*i*_),

$$r_{\mathrm{CoM}}=\sum_{i}w_{i}r_{i}$$

where *w*_*i*_ are weight coefficients adapted from Winter [20] (Table S1). Thereafter, CoM velocity (*v*_CoM_) and acceleration (*a*_CoM_) were obtained from the CoM trajectory using the central‐difference scheme, and instantaneous CoM mechanical power was computed as follows:

$$m\left(a_{\mathrm{CoM}}-g\right)\cdot v_{\mathrm{CoM}}$$

with *m* denoting body mass and *g* the gravitational acceleration vector.

**FIGURE 2:**
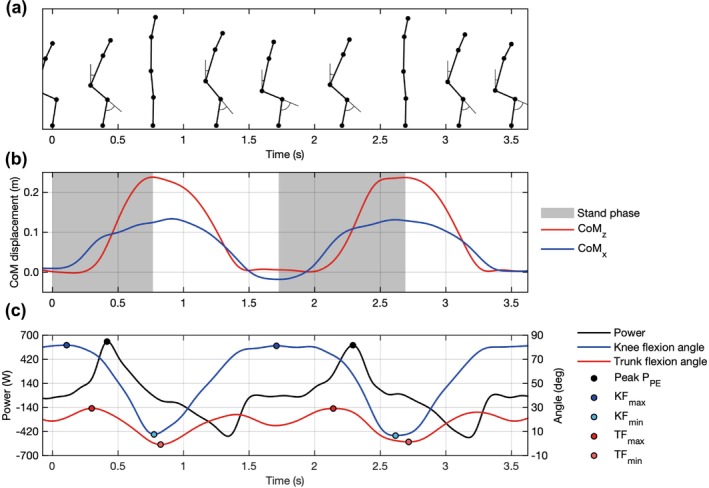
Schematic of pose‐estimated kinematic variables for an example participant performing two STS repetitions. (a) Stick‐figure from 2D pose estimation at representative time points along the common x‐axis; joints are plotted at the vertex, shoulder, hip, knee and ankle. (b) Centre‐of‐mass trajectory with stand phases indicated as grey shade. (c) STS power (black solid line), knee flexion angle (blue solid line) and trunk flexion angle (red solid line). Peaks are annotated as follows: peak STS power (Peak P_PE_, black filled circles); KF_max_ (dark blue); KF_min_ (light blue); TF_max_ (red); TF_min_ (orange).

Following [21], an STS repetition was defined as the interval between consecutive local minima of the anteroposterior CoM position (CoM_x_) and the stand phase as the period from repetition onset to the peak vertical CoM position (CoM_z_). Kinematic parameters were defined as follows and are illustrated in Figure 2.

1. Peak P_PE_: The positive peak of STS power during each stand phase.
2. T_peak_: The time from the start of the test to each peak P_PE_.
3. Knee flexion angle: Angle between the hip–knee and knee–ankle segments; 0° indicates full extension.
4. KF_max_/KF_min_: The maximum and minimum knee flexion angles within a cycle.
5. Trunk flexion angle: Angle between the shoulder–hip segment and the z‐axis.
6. TF_max_/TF_min_: The maximum and minimum trunk flexion angles within a cycle.

### 2.5 Construct Validation

In the validation subgroup (*n* = 20), participants performed the 30‐s sit‐to‐stand (STS) task while concurrent data were collected using, 2D pose estimation, 3D optical motion capture (Motion Analysis Corp., Santa Rosa, CA, USA), and force‐plate recordings (Kistler 9260AA6; Kistler Instrumente AG, Winterthur, Switzerland) were acquired concurrently (Figure 1b). The motion capture and force plate systems were synchronized within Orthotrak 6.6 (Motion Analysis Corporation) and sampled at 120 Hz. A full‐body Helen Hayes marker set, consisting of 31 reflective markers, was used for motion capture [22], and a single force plate was placed under both feet. Participants were barefoot and wore a swimming cap and shorts to ensure accurate measurement of ground‐reaction force (GRF) and marker trajectories. From the 3D motion‐capture data, we identified the bilateral hip, knee and ankle joint centres, as well as the bilateral acromial tips, lateral epicondyles and wrist landmarks (the midpoint between the radial and ulnar styloid processes). Trajectories were low‐pass filtered using a fourth‐order Butterworth filter with a cut‐off frequency of 4 Hz. The centre of mass (CoM) was calculated as a weighted average of these landmarks, employing the same formulation used for pose estimation. CoM velocity ($v_{\mathrm{CoM}}$) was obtained using central differences, and reference STS power was calculated as $P_{\mathrm{ref}}=\mathrm{GRF}\cdot v_{\mathrm{CoM}}$. Joint angles were computed in the sagittal (x–z) plane. The knee angle was calculated bilaterally and averaged across limbs. All other kinematic variables were derived using procedures used for pose‐estimation data.

### 2.6 Muscle Mass and Strength Measurements

Bioelectrical impedance analysis was performed using the InBody 770 (InBody Co., Seoul, Republic of Korea) to measure appendicular muscle mass. This measurement was then converted to the appendicular skeletal muscle index (ASMI) by dividing by squared height. Body mass and height were accurately measured, to the nearest 0.1 cm and 0.1 kg, respectively, using the scale and stadiometer integrated into the BIA device. Muscle strength was assessed through handgrip strength and isometric knee extensor strength. Handgrip strength was measured with a Smedley Hand Dynamometer (Grip‐D, TKK5101; Takei Scientific Instruments, Niigata, Japan), following established protocols from previous literature [23]. Maximal isometric knee extensor strength was measured in a sitting position using a custom‐made dynamometer chair with the knee positioned at a 90° angle, in accordance with previous literature [23]. The torque for knee extension (Nm) was calculated by multiplying the maximal strength (N) by the length of the lever arm (m).

### 2.7 Physical Performance Assessments

Physical performance was assessed using the SPPB, usual walking speed, and the TUG test. The SPPB was administered following a standardized protocol [24]. It consists of three components: a balance test, a 4‐m walk test and the 5x‐STS. Each component was scored on a scale from 0 (*unable to complete*) to 4 (*best performance*), resulting in a total score ranging from 0 to 12, with higher scores indicating better physical performance. For the usual walking speed assessment, participants traversed a 10‐m walkway at a self‐selected comfortable pace. Timing was conducted with a digital stopwatch over the central 6 m, following a 2‐m acceleration phase and preceding a 2‐m deceleration phase. Gait speed (m/s) was calculated by dividing the 6 m by the timed interval [25]. In the TUG test, participants stood up from a standard chair with a height of 46 cm, walked to and turned around a cone placed 3 m away, then returned to the chair and sat down at their normal, comfortable pace. Timing began with the verbal cue ‘go’ and stopped upon full seating [25]. TUG velocity (m/s), calculated as *6/TUG time*, was used to harmonize directionality with other metrics.

### 2.8 Statistical Analysis

Statistical analyses were conducted using SPSS version 29.0 (SPSS Inc., Chicago, Illinois), and kinematic data analyses were performed using MATLAB R2023b. The level of significance was set at a *p*‐value of < 0.05. Clinical characteristics are presented as mean ± standard deviation (SD). Results for pose‐estimated variables are summarized as grand mean ± between‐subject SD and within‐subject SD. Sex differences were examined using an independent‐samples *t*‐test.

To validate the pose‐estimation results, the STS power, knee flexion angle and trunk flexion angle were visualized by ensemble plots of the grand mean of participant‐level mean ± between‐subject SD. To evaluate overall accuracy, the root mean squared errors (RMSE) between the pose‐estimation and the reference methods were calculated. Additionally, the correlations between peak values of pose‐estimation variables and the reference method were assessed using univariate linear regressions based on participant‐level means. Agreement between peak P_PE_, KF_max_, KF_min_, TF_max_ and TF_min_, and the corresponding reference measures were assessed with Bland–Altman analyses and intraclass correlation coefficients using a two‐way random‐effects, absolute‐agreement and single‐measure model (ICC[2, 1]). ICC values were interpreted as poor (< 0.50), moderate (0.50–0.75), good (0.75–0.90) and excellent (> 0.90).

The associations between STS metrics (peak P_PE_, 5x‐STS, 30‐s STS) and physical performance (usual walking speed, TUG velocity, SPPB), muscle strength (knee extensor and handgrip strength) and muscle mass (ASMI) were assessed using Pearson's correlation. Following [26], we interpreted Pearson's correlation coefficient as weak (|*r*| < 0.30), moderate (0.30 ≤ |*r*| ≤ 0.60) and strong (|r| > 0.60). To evaluate the potential impact of confounders such as sarcopenia status and comorbidities on STS metrics, we employed analysis of covariance models for Peak P_PE_, 5x‐STS and 30‐s STS. Sarcopenia status was categorized according to AWGS 2019 and EWGSOP2 criteria, while comorbidities were classified based on their quantity (0, 1 or ≥ 2) and type (including diabetes, hypertension, dyslipidaemia, heart disease, a history of cancer and any thyroid, liver, lung or kidney disease). The non‐sarcopenia and no‐comorbidity group served as the reference. All models were adjusted for sex and body mass, and we calculated adjusted mean differences (β ± SE) relative to the reference group. Given the significant associations between AWGS 2019 sarcopenia status and STS metrics, we also calculated Pearson correlation coefficients for a combined subgroup of participants with probable sarcopenia, sarcopenia, or severe sarcopenia, as defined by AWGS 2019, examining the relationships between STS metrics and clinical measurements.

To assess the influence of within‐subject variability on peak P_PE_, we used sex‐stratified linear mixed‐effects regression models. Participants were modelled as a random effect, and KF_max_, KF_min_, TF_max_, TF_min_ and T_peak_ were included as fixed effects. To isolate within‐participant effects, the dependent variable (Peak P_PE_) was normalized to each participant's mean and expressed as percent change [(value − participant mean)/participant mean × 100%]; angular predictors (KF_max_, KF_min_, TF_max_, TF_min_) were subject‐mean centred, and T_peak_ was entered on its original scale. Due to significant sex‐by‐kinematics interactions, analyses were conducted separately for men and women.

## 3 Results

### 3.1 Clinical Characteristics of Study Participants

Table 1 presents the characteristics of the participants. A total of 129 community‐dwelling elderly individuals, with a mean age of 71.8 years, were included in the study, of which 58% (*n* = 75) were women. Compared to females, males were significantly older (73.0 vs. 71.0 years, *p* = 0.018), taller (165.7 vs. 154.4 cm, *p* < 0.001) and heavier (65.8 vs. 56.5 kg, *p* < 0.001). Additionally, males had a higher ASMI (7.60 vs. 6.17 kg/m^2^, *p* < 0.001), greater knee extensor strength (127.9 vs. 75.1 Nm, *p* < 0.001) and increased handgrip strength (31.5 vs. 19.1 kg, *p* < 0.001), as well as a faster usual walking speed (1.46 vs. 1.38 m/s, *p* = 0.019). However, no significant differences were observed in BMI, TUG, TUG velocity, 5x‐STS or 30‐s STS (*p* > 0.05 for all).

**TABLE 1:** Subject characteristics.

Characteristic	Total (*n* = 129)	Males (*n* = 54)	Females (*n* = 75)	*p*
Age (years)	71.8 ± 4.5	73.0 ± 5.0	71.0 ± 3.8	0.012
Height (cm)	159.1 ± 7.2	165.7 ± 3.5	154.4 ± 5.1	< 0.001
Body mass (kg)	60.4 ± 9.0	65.8 ± 8.2	56.5 ± 7.4	< 0.001
BMI (kg/m^2^)	23.8 ± 2.9	24.0 ± 2.8	23.7 ± 3.0	0.642
ASMI (kg/m^2^)	6.77 ± 0.91	7.60 ± 0.58	6.17 ± 0.56	< 0.001
Knee extensor strength (Nm)	97.2 ± 39.4	127.9 ± 37.5	75.1 ± 22.2	< 0.001
Handgrip strength (kg)	24.3 ± 7.7	31.5 ± 5.5	19.1 ± 4.1	< 0.001
Usual walking speed (m/s)	1.41 ± 0.19	1.46 ± 0.19	1.38 ± 0.19	0.019
TUG (seconds)	8.85 ± 1.66	8.86 ± 1.74	8.85 ± 1.61	0.955
TUG velocity (m/s)	0.70 ± 0.12	0.70 ± 0.11	0.70 ± 0.13	0.889
SPPB (points)	11.5 ± 0.8	11.5 ± 1.0	11.6 ± 0.7	0.519
5x‐STS (seconds)	10.15 ± 2.48	10.22 ± 2.87	10.11 ± 2.18	0.797
30‐s STS (*n*)	16.0 ± 4.4	16.1 ± 4.5	15.9 ± 4.5	0.782
Sarcopenia status by AWGS 2019				0.085
Probable sarcopenia, *n* (%)	60 (46.5%)	21 (38.9%)	39 (52.0%)	
Sarcopenia, *n* (%)	10 (7.8%)	1 (1.9%)	9 (12.0%)	
Severe sarcopenia, *n* (%)	2 (1.6%)	0	2 (2.7%)	
Sarcopenia status by EWGSOP 2				0.406
Probable sarcopenia, *n* (%)	33 (25.6%)	15 (27.8%)	18 (24.0%)	
Sarcopenia, *n* (%)	5 (3.9%)	1 (1.9%)	4 (5.3%)	
Severe sarcopenia, *n* (%)	0	0	0	
Comorbidities				
Hypertension, *n* (%)	58 (45.0%)	22 (40.7%)	36 (48.0%)	0.414
Dyslipidaemia, *n* (%)	58 (45.0%)	17 (31.5%)	41 (54.7%)	0.009
Diabetes, *n* (%)	25 (19.4%)	13 (24.1%)	12 (16.0%)	0.252
Heart disease, *n* (%)	12 (9.3%)	6 (11.1%)	6 (8.0%)	0.548
Previous history of cancer, *n* (%)	12 (9.3%)	2 (3.7%)	10 (13.3%)	0.063
Thyroid disease, *n* (%)	6 (4.7%)	1 (1.9%)	5 (6.7%)	0.400
Liver disease, *n* (%)	5 (3.9%)	2 (3.7%)	3 (4.0%)	1.000
Chronic lung diseases, *n* (%)	2 (1.6%)	0	2 (2.7%)	0.509
Kidney disease, *n* (%)	1 (0.8%)	0	1 (1.3%)	1.000
Number of comorbidities				0.193
0	37 (28.7%)	19 (35.2%)	18 (24.0%)	
1	35 (27.1%)	16 (29.6%)	19 (25.3%)	
2 or more	57 (44.2%)	19 (35.2%)	38 (50.7%)	

*Note:* Values are mean ± SD. *p* values were derived from unpaired Student's *t*‐tests (continuous variables) and *χ* ^2^ or Fisher's exact tests (categorical variables) comparing males and females.

Abbreviations: 30‐s STS, 30‐s sit‐to‐stand test; 5x‐STS, 5‐times sit‐to‐stand test; ASMI, appendicular skeletal muscle index; AWGS 2019, Asian Working Group for Sarcopenia 2019; BMI, body mass index; EWGSOP2, European Working Group on Sarcopenia in Older People 2; KES, knee extensor strength; SD, standard deviation; SPPB, Short Physical Performance Battery; TUG, Timed Up and Go test.

Using the AWGS 2019 criteria, 60 participants (46.5%) were classified as having probable sarcopenia, 10 (7.8%) as having sarcopenia and 2 (1.6%) as having severe sarcopenia. According to the EWGSOP2 definition, 33 participants (25.6%) met the criteria for probable sarcopenia, and 5 (3.9%) were classified as having sarcopenia, with no cases of severe sarcopenia identified. In terms of comorbidities, hypertension and dyslipidaemia were the most prevalent, affecting 45.0% of participants each, followed by diabetes at 19.4%. Heart disease and a history of cancer were present in 9.3% of participants, while other chronic conditions, such as lung, liver, thyroid and kidney disease, were uncommon, affecting less than 5%.

### 3.2 Validation of Pose‐Estimation Variables

Among the 20 participants in the validation subgroup, 14 (70%) were women. Clinical characteristics are summarized in Table S2. Ensemble trajectories from the pose‐estimation model closely aligned with those from the reference method for STS power, knee flexion angle, and trunk flexion angle (see Figure S1). The RMSE values were 66.6 ± 55.1 W for STS power, 5.9° ± 3.7° for knee flexion angle and 4.0° ± 2.1° for trunk flexion angle.

**FIGURE 3:**
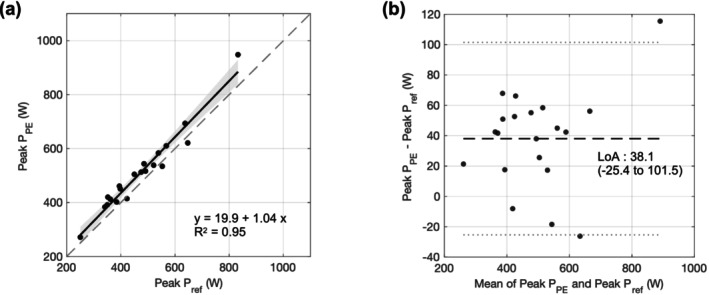
Validation of peak STS power. (a) Scatter plot of peak STS power from pose estimation (Peak P_PE_) versus the reference method (Peak P_ref_). Each dot represent the participant‐level means. The black solid line shows the fitted regression; the shaded band denotes the 95% confidence interval. (b) Bland–Altman plot of participant‐level means (pose − reference). The black dashed line indicates the mean bias; grey dotted lines indicate the upper and lower 95% limits of agreement.

The pose‐estimation algorithm yielded higher peak STS power, smaller peak knee flexion angles and larger peak trunk flexion angles than the reference (Peak P_PE_: 510.7 vs. 472.7 W, *p* < 0.001; KF_max_ 83.1° vs. 85.3°, *p* = 0.003; KF_min_ 9.4° vs. 11.7°, *p* < 0.001; TF_max_ 27.5° vs. 24.4°, *p* < 0.001; TF_min_ −0.2° vs −2.6°, *p* < 0.001). Bland–Altman analyses of subject‐level means showed significant constant biases for peak P_PE_ (mean bias 38.1 W, 95% CI 22.9–53.2), KF_max_ (mean bias −2.2°, 95% CI −3.5° to −0.8°), KF_min_ (mean bias −2.3°, 95% CI −3.4 to −1.1) and TF_min_ (mean bias 2.4°, 95% CI 1.5–3.3). For TF_max_, significant proportional bias was present (difference = −1.8 + 0.19 × Mean; 95% CI for intercept −6.8 to 3.2, 95% CI for slope 0.00–0.37). Despite significant differences, agreement between methods was high by ICC. Peak P_PE_ showed excellent agreement with ICC = 0.94, whereas KF_max_, KF_min_, TF_max_ and TF_min_ showed good agreement (KF_max_: 0.84; KF_min_: 0.83; TF_max_: 0.83; TF_min_: 0.89). Convergent validity was further supported by a high coefficient of determination in the participant‐mean regressions of pose estimation on the reference (peak P_PE_, *R* ^2^ = 0.95; KF_max_, *R* ^2^ = 0.80; KF_min_, *R* ^2^ = 0.80; TF_max_, *R* ^2^ = 0.87; TF_min_, *R* ^2^ = 0.91). (Figures 3, S2 and S3 and Table S3)

### 3.3 Pose‐Estimation Variables

The kinematic parameters and peak P_PE_ derived from pose estimation are shown in Table 2. On average, participants demonstrated a peak P_PE_ of 423.8 W; the peak knee flexion angles were 87.4° (KF_max_) and 8.0° (KF_min_), and trunk flexion angles were 25.9° (TF_max_) and −2.7° (TF_min_). Males exhibited significantly higher peak P_PE_ (513.8 W vs. 359.1 W, *p* < 0.001) and KF_max_ (90.7° vs. 85.1°, *p* < 0.001) compared to females. However, no significant differences were found in KF_min_, TF_max_ and TF_min_.

**TABLE 2:** Variables derived from 2D pose estimation of the 30‐s STS.

Variables	Total (*n* = 129)		Male (*n* = 54)		Female (*n* = 75)		*p*
	$\bar{\mu }$ ± SD_between_	SD_within_	$\bar{\mu }$ ± SD_between_	SD_within_	$\bar{\mu }$ ± SD_between_	SD_within_	
Peak P_PE_ (W)	423.8 ± 126.1	39.0	513.8 ± 116.3	46.2	359.1 ± 87.5	32.8	< 0.001
KF_max_ (°)	87.4 ± 6.7	1.9	90.7 ± 6.5	1.8	85.1 ± 5.7	2.0	< 0.001
KF_min_ (°)	8.1 ± 5.7	3.6	6.8 ± 5.4	3.2	9.1 ± 5.7	3.8	0.024
TF_max_ (°)	25.9 ± 6.5	2.6	26.5 ± 6.2	2.6	25.4 ± 6.6	2.6	0.380
TF_min_ (°)	−2.7 ± 6.0	2.7	−2.4 ± 5.5	2.7	−2.9 ± 6.4	2.7	0.689

*Note:* Values are $\bar{\mu }$ (grand mean) ± SD_between_ (between‐subject SD). SD_within_ denotes the within‐subject SD across repetitions. *p* values are from independent Student's *t*‐tests comparing males versus females.

Abbreviations: KF, knee flexion angle; Peak P_PE_, pose‐estimation–derived peak STS power; SD_between_, between‐subject standard deviation; SD_within_, within‐subject standard deviation; TF, trunk flexion angle.

### 3.4 Correlation Between STS Metrics and Physical Performance, Muscle Mass and Muscle Strength

Table 3 presents Pearson's correlation coefficients for the relationships between STS metrics and physical performance, muscle strength, and muscle mass. Peak P_PE_ exhibited strong correlations with muscle strength indices, such as knee extensor strength (*r* = 0.64, *p* < 0.001) and handgrip strength (*r* = 0.64, *p* < 0.001), as well as with ASMI (*r* = 0.70, *p* < 0.001). In contrast, the associations with physical performance were weak, including usual walking speed (*r* = 0.19, *p* = 0.030), TUG velocity (*r* = 0.05, *p* = 0.575) and SPPB (*r* = 0.16, *p* = 0.075). The 5x‐STS time showed moderate to strong associations with poorer performance outcomes: usual walking speed (*r* = −0.31, *p* < 0.001), TUG velocity (*r* = −0.59, *p* < 0.001) and SPPB (*r* = −0.79, *p* < 0.001). However, it was not significantly correlated with strength or muscle mass (*p* > 0.05). Similarly, the 30‐s STS repetitions positively correlated with all physical performance measures: usual walking speed (*r* = 0.19, *p* = 0.032), TUG velocity (*r* = 0.42, *p* < 0.001) and SPPB (*r* = 0.52, *p* < 0.001) but did not show significant correlations with muscle strength or mass indices.

**TABLE 3:** Pearson correlations between STS metrics and clinical measures.

	Peak P_PE_		5x‐STS		30‐s STS	
	*r*	*p*	*r*	*p*	*r*	*p*
Physical performance						
Usual walking speed	0.19	0.030	−0.31	< 0.001	0.19	0.032
TUG velocity	0.05	0.575	−0.59	< 0.001	0.42	< 0.001
SPPB	0.16	0.075	−0.79	< 0.001	0.52	< 0.001
Muscle strength						
Knee extensor strength	0.64	< 0.001	−0.17	0.059	0.17	0.057
Handgrip strength	0.64	< 0.001	−0.08	0.353	0.10	0.248
Muscle mass						
ASMI	0.70	< 0.001	0.02	0.817	0.05	0.601

Abbreviations: 5x‐STS, five‐times sit‐to‐stand test; 30‐s STS, 30‐s sit‐to‐stand test; ASMI, appendicular skeletal muscle index; Peak P_PE_, pose‐estimation–derived peak STS power; SPPB, Short Physical Performance Battery; TUG, Timed Up and Go.

The potential effects of sarcopenia status and comorbidities on STS metrics were analysed using ANCOVA across subgroups, adjusting for sex and body mass. The models indicated no significant impact from the number of comorbidities, any specific comorbidity or sarcopenia status as defined by EWGSOP2 on any STS metric (see Table S4). In contrast, probable sarcopenia as defined by AWGS 2019 was linked to lower Peak P_PE_ (β = −44.1 ± 14.9) and fewer repetitions in the 30s‐STS (β = −1.7 ± 0.8; both *p* < 0.05), while the difference in the 5x‐STS was small and not statistically significant (β = 0.58 ± 0.46). To determine whether the significant confounder, sarcopenia status defined by AWGS 2019, affected the correlation patterns, we recalculated correlations within the combined subgroup of probable sarcopenia, sarcopenia, and severe sarcopenia (see Table S5). The correlation patterns were generally consistent with those observed in the overall sample: Peak P_PE_ remained strongly correlated with knee extensor strength (*r* = 0.67, *p* < 0.001), handgrip strength (*r* = 0.65, *p* < 0.001) and ASMI (*r* = 0.76, *p* < 0.001). Meanwhile, 5x‐STS and 30‐s STS demonstrated stronger correlations with physical performance measures, including TUG velocity (*r* = −0.62 and 0.46, respectively; both *p* < 0.001) and SPPB (*r* = −0.79 and 0.56, respectively; both *p* < 0.001).

### 3.5 Posture and Fatigue as Predictors of Within‐Subject Variability in Peak P_PE_

Using sex‐stratified linear mixed‐effects models with within‐subject normalized predictors, changes in joint angles and timing explained variation in ΔPeak P_PE_ (%). Among men (*n* = 54), ΔKF_min_ was inversely associated with ΔPeak P_PE_ (B = −0.25, *p* = 0.009), and ΔTF_min_ was positively associated (B = 0.35, *p* = 0.003); associations with ΔKF_max_, ΔTF_max_ and T_peak_ were not significant. Among women (*n* = 75), ΔKF_min_ showed a stronger inverse association (B = −0.67, *p* < 0.001), ΔKF_max_ was positively associated (B = 0.36, *p* = 0.005) and T_peak_ was inversely associated with ΔPeak P_PE_ (B = −0.25, *p* < 0.001), whereas ΔTF_max_ and ΔTF_min_ were not significant. The fixed‐effects explained a modest proportion of variance (marginal *R* ^2^ = 0.024 in men; 0.138 in women) (Table 4 and Figures S4 and S5)

**TABLE 4:** Factors associated with within‐subject variability in peak P_PE_: sex‐stratified linear mixed‐effects models.

Group	Dependent variable	Variables	B (95% CI)	*p*
Male (*n* = 54)	ΔPeak P_PE_ (%)	ΔKF_max_	0.28 (−0.06 to 0.61)	0.102
		ΔKF_min_	−0.25 (−0.45 to −0.06)	0.009
		ΔTF_max_	0.09 (−1.26 to 1.43)	0.921
		ΔTF_min_	0.35 (0.12 to 0.58)	0.003
		T_peak_	−0.03 (−0.11 to 0.05)	0.441
Female (*n* = 75)	ΔPeak P_PE_ (%)	ΔKF_max_	0.36 (0.11 to 0.61)	0.005
		ΔKF_min_	−0.67 (−0.80 to −0.54)	< 0.001
		ΔTF_max_	−0.03 (−0.25 to 0.18)	0.762
		ΔTF_min_	0.12 (−0.06 to 0.18)	0.180
		T_peak_	−0.25 (−0.31 to −0.19)	< 0.001

*Note:* Entries are fixed‐effect coefficients (B) with 95% confidence intervals and two‐sided *p*‐values. Predictors were normalized within participant (mean‐centred) for KF_max_, KF_min_, TF_max_ and TF_min_; Δ denotes within‐subject deviation. The dependent variable, ΔPeak P_PE_ (%), is the percent deviation of Peak P_PE_ from each participant's mean. Marginal *R* ^2^ (fixed effects only): 0.024 in men and 0.138 in women.

Abbreviations: CI, confidence interval; KF, knee flexion angle; Peak P_PE_, pose‐estimation–derived peak STS power; TF, trunk flexion angle; T_peak_, time to peak power.

## 4 Discussion

In this cross‐sectional study of 129 community‐dwelling older adults, we investigated whether STS metrics reflect primarily reflect muscle strength or physical performance. To validate the pose estimation algorithm, we compared it with motion capture and force‐plate data in a subgroup of 20 participants. The agreement of the pose estimated variables was high, demonstrating strong subject‐mean regressions. In the full cohort, peak P_PE_ aligned more closely with muscle strength and mass than with performance measures, while the 5x‐STS and 30‐s STS tracked physical performance more strongly than muscle strength. The pattern of association was similar for combined subgroup of possible sarcopenia, sarcopenia, and severe sarcopenia, defined by AWGS 2019. Posture‐related joint angles significantly contributed to within‐subject variability in peak P_PE_. In women only, the fatigue proxy T_peak_ was inversely associated with within‐subject variability in peak P_PE_.

To our knowledge, the literature on the concurrent associations between STS power, muscle strength, and physical performance outcomes in a single cohort of community‐dwelling older adults is limited. A previous study [27] examined these associations using leg‐press tasks, finding a strong correlation between leg‐press power and leg‐press (*r* = 0.89), with leg‐press power accounting for 12%–45% of the variance in physical performance. Other studies have similarly high correlations between knee extensor strength and STS power (Takai et al. [28] *r* = 0.73; Lindemann et al. [14] *r* = 0.68). Consistent with this, our peak P_PE_ showed strong correlations with muscle strength indicators (knee extensor strength, *r* = 0.64; handgrip strength, *r* = 0.64), while associations with physical performance were weaker (usual walking speed, *r* = 0.19; TUG velocity, *r* = 0.05; SPPB, *r* = 0.16). In contrast, the 5x‐STS time and 30‐s STS repetitions demonstrated higher correlations with physical performance than with muscle strength in our study, aligning with findings by X. S. Yee et al. [29]. These patterns provide context for current guideline classifications. The AWGS 2019 designates the STS test as a measure of physical performance measure, while EWGSOP2 classifies it measure of muscle strength. In light of previous research [29], our results align more closely with the AWGS 2019 framework: The STS is more likely to reflect physical performance when operationalized via repetition/time metrics, whereas power‐based STS measures are more indicative of muscular strength.

In our preliminary validation analysis, the peak P_PE_ demonstrated a very strong association with the reference method (*R* ^2^ = 0.95) and excellent agreement (ICC = 0.94). However, a statistically significant mean bias of +38.1 W (95% CI 22.9–53.2) was observed. This bias likely arises from the effect of height‐only length scaling (without segment or scene calibration) and the misalignment between pose estimation key points and motion‐capture joint centres. Nevertheless, the absolute magnitude of this bias was smaller than that reported in a prior comparison of IMU sensors with motion capture and force plate (−58 W) [16], which supports the strength of the present study. The root mean square errors for joint angles between pose estimation and motion capture were 5.9° for knee flexion and 4.0° for trunk flexion, which are comparable to those reported for marker‐less pose estimation systems, around 5.8° for lower‐limb joint angles across various tasks [30]. However, this validation was performed in a subgroup of only 20 participants, thus providing primarily a technical benchmark for agreement. The small sample size may restrict the precision and generalizability of these estimates. To establish the robustness of these findings, additional studies involving larger, independent cohorts are required.

Sex‐stratified linear mixed‐effects models indicated that posture and the time since the start of 30‐s STS significantly contribute to within‐subject variability in peak power. The fixed effects explained a larger share of variability in women than in men (marginal *R* ^2^ = 0.138 vs. 0.024). KF_min_ was a significant predictor for both sexes: each 1° increase in KF_min_ was associated with a 0.67% decrease in peak P_PE_ for women and a 0.25% decrease for men. In women, greater KF_max_ was associated with higher peak P_PE_, consistent with evidence that a lower seat increases centre of mass (CoM) velocity and mechanical demand, resulting in larger ground reaction forces (GRF) and higher STS power [31, 32]. In men, greater TF_min_ was associated with higher peak P_PE_, suggesting that retaining some forward trunk flexion at the end of the rise may be more advantageous for power generation than fully straightening the trunk. While conventional STS protocols typically target KF_max_ as 90° and KF_min_ as 0°, our results allow more quantitative guidance. Using an arbitrary ±5% tolerance, the within‐person bands can be suggested as women—ΔKF_min_ ± 7.5°, ΔKF_max_ ± 13.9°, men—ΔKF_min_ ± 20.0° and ΔTF_min_ ± 14.3°. These bands represent allowable deviations around each individual's mean posture and can support tighter standardization of STS testing. Notably, in women, T_peak_ was a significant predictor and alighted with a notable decline in peak P_PE_ during the 30‐s STS. This pattern may reflect greater susceptibility to fatigue in women, although the influence of effort or pacing component cannot be ruled out.

Recent studies have increasingly employed pose estimation algorithms to quantify the STS test. In one study, at‐home smartphone videos were analysed with pose estimation to derive STS time and maximal trunk flexion angle, showed predictive utility for physical health status and osteoarthritis [33]. In another study, a smartphone application computed STS power from rise time and femoral length, demonstrated good diagnostic performance for sarcopenia detection [34]. A distinctive feature of the present study is the biomechanical analysis of STS movements from video using pose estimation to quantify STS power and kinematic parameters. This method has proven accurate when compared to reference measurements from motion capture and a force plate. Looking ahead, this approach could facilitate scalable, home‐based screening for sarcopenia and frailty, enable longitudinal monitoring of fall risk, and inform adaptive interventions to prevent mobility decline.

### 4.1 Limitations

First, participants had relatively preserved physical performance (mean SPPB 11.5), so the observed associations between STS metrics and physical performance or muscle strength may not generalize to populations with greater disability or multimorbidity. In these populations, STS power is expected to change more sensitively than muscle strength [35], so the strength–power association may be steeper than that observed in our study. By contrast, correlations between 30‐s STS or 5x‐STS and global physical performances are likely to be more variable as both may decline at similar rate. Second, CoM was approximated from weighted key points, which may not capture individual body shape/composition, and our analysis assumed right–left symmetry, both of which may contribute to measurement error. Third, the sample size (*n* = 129) was modest, which may limit the precision of our estimates and our ability to detect smaller effects. Therefore, our findings should be validated in larger, independent cohorts, including studies aimed at establishing clinically applicable STS power cut‐off values.

## 5 Conclusions

In this cross‐sectional study of 129 older adults, pose‐estimated peak STS power was more closely aligned with muscular strength, while the 5x‐STS and 30‐s STS measures tracked overall functional performance and were consistent with the AWGS 2019 framework. Pose‐estimated peak power and joint angles showed strong agreement with motion‐capture and force‐plate references in the preliminary validation. Joint angles impacted the variability of peak STS power within subjects for both men and women, although fatigue primarily affected women. These findings enhance the interpretation of STS metrics and support the use of pose estimation as a practical tool for longitudinal fall‐risk monitoring, home‐based sarcopenia screening, and remote functional assessment.

## Funding

This research was supported by a grant from the Patient‐Centered Clinical Research Coordinating Center (PACEN) funded by the Ministry of Health & Welfare, Republic of Korea (grant no. HC21C0064).

## Ethics Statement

The study was reviewed and approved by the Institutional Review Boards of the Seoul National University Hospitals (IRB No. 2104‐125‐1213). All participants provided written informed consent.

## Conflicts of Interest

The authors declare no conflicts of interest.

## Supporting information


**Table S1:** Key points, weight coefficients and perspective corrections for pose estimation.
**Table S2:** Subject characteristics for validation subgroup (*n* = 20).
**Table S3:** Agreement of pose‐estimated peak power and joint angles with the reference method (participant‐level means).
**Table S4:** Differences in sit‐to‐stand metrics according to sarcopenia status and comorbidities.
**Table S5:** Pearson correlations between sit‐to‐stand metrics and clinical measures among participants with probable sarcopenia, sarcopenia or severe sarcopenia (AWGS 2019 criteria).

**Figure S1:** Ensemble trajectories for (a) STS power, (b) knee flexion angle and (c) trunk flexion angle. Blue solid line: pose estimation; red solid line: reference; grey dashed line: pointwise RMSE over the normalized cycle; curves: grand mean across participants; grey shaded band: grand mean ± SD_between_.
**Figure S2:** Validation of peak joint angles from pose estimation. Participant‐level mean (a) KF_max_, (b) KF_min_, (c) TF_max_ and (d) TF_min_ from pose estimation plotted against motion capture. Black solid line: fitted regression; grey shaded band: 95% confidence interval.
**Figure S3:** Bland–Altman plots for participant‐level means of (a) KF_max_, (b) KF_min_, (c) TF_max_ and (d) TF_min_. The black dashed line marks the mean bias, and the grey dotted lines indicate the upper and lower 95% limits of agreement.
**Figure S4:** Linear mixed‐effects model for male. Scatterplots of within‐subject associations between ΔPeak P_PE_ (%) and (a) ΔKF_max_, (b) ΔKF_min_, (c) ΔTF_max_, (d) ΔTF_min_ and (e) T_peak_. Black solid line: fixed‐effect fit; grey shaded band: 95% CI for fixed effect.
**Figure S5:** Linear mixed‐effects model for female. Scatterplots of within‐subject associations between ΔPeak P_PE_ (%) and (a) ΔKF_max_, (b) ΔKF_min_, (c) ΔTF_max_, (d) ΔTF_min_ and (e) T_peak_. Black solid line: fixed‐effect fit; grey shaded band: 95% CI for fixed effect.
