# Correction to ‘Sit‐ to‐ Stand Power From 2D Pose Estimation as an Indicator of Muscle Strength in Older Adults’

**DOI:** 10.1002/jcsm.70258

**Published:** 2026-04-15

W. Hwang , D. Shim , J. Kim , et al., “Sit‐to‐Stand Power From 2D Pose Estimation as an Indicator of Muscle Strength in Older Adults,” Journal of Cachexia, Sarcopenia and Muscle 17, no. 1 (2026): e70208, 10.1002/jcsm.70208.41588557 PMC12834695

In the Funding section, a grant number has been omitted. The correct Funding statement is shown below:

This research was supported by a grant from the Patient‐ Centered Clinical Research Coordinating Center (PACEN) funded by the Ministry of Health & Welfare, Republic of Korea (Grant Nos. HC21C0064 and RS‐2025‐KH02222172).

We apologize for this error.

