# Supplementary material for: Sit‐to‐Stand Power From 2D Pose Estimation as an Indicator of Muscle Strength in Older Adults
Source: J Cachexia Sarcopenia Muscle. 2026 Jan 26;17(1):e70208. doi: 10.1002/jcsm.70208 (PMC12834695; doi:10.1002/jcsm.70208)
Supplement: Supplementary file 1 — Table S1: Key points, weight coefficients and perspective corrections for pose estimation. Table S2: Subject characteristics for validation subgroup (n = 20). Table S3: Agreement of pose‐estimated peak power and joint angles with the reference method (participant‐level means). Table S4: Differences in sit‐to‐stand metrics according to sarcopenia status and comorbidities. Table S5: Pearson correlations between sit‐to‐stand metrics and clinical measures among participants with probable sarcopenia, sarcopenia or severe sarcopenia (AWGS 2019 criteria). [file JCSM-17-e70208-s002.docx]

**Table S1**. keypoints, weight coefficients, and perspective corrections for pose estimation.

| **Keypoints** | **Weight coefficients** | **Perspective correction** |
| --- | --- | --- |
| Vertex | 0.081000 | 1 |
| Shoulder | 0.087098 | 1 – 0.5 x 0.259 x h/L |
| Elbow | 0.019204 | 1 – 0.5 x 0.259 x h/L |
| Wrist | 0.015004 | 1 – 0.5 x 0.259 x h/L |
| Hip | 0.234984 | 1 – 0.5 x 0.191 x h/L |
| Knee | 0.066940 | 1 – 0.5 x 0.191 x h/L |
| Ankle | 0.036360 | 1 – 0.5 x 0.191 x h/L |

Weight coefficients were modified from Winter (2009). h/L is the ratio of participant’s height (h) to horizontal camera–participant distance (L).

**Table S2** Subject characteristics for validation subgroup. (n = 20)

| **Characteristic** | **Validation subgroup (n=20)** |
| --- | --- |
| Age (years) | 73.5 ± 4.4 |
| Sex (M: F) | 6: 14 |
| Height (cm) | 159.1 ± 6.9 |
| Body mass (kg) | 60.9 ± 8.5 |
| BMI (kg/m^2^) | 24.0 ± 2.5 |
| ASMI (kg/m^2^) | 6.72 ± 0.90 |
| Knee extensor strength (Nm) | 86.2 ± 28.3 |
| Handgrip strength (kg) | 22.8 ± 5.7 |
| Usual walking speed (m/s) | 1.30 ± 0.15 |
| TUG (seconds) | 9.69 ± 1.68 |
| TUG velocity (m/s) | 0.64 ± 0.11 |
| SPPB (points) | 11.6 ± 0.8 |
| 5x-STS (seconds) | 10.04 ± 2.30 |
| 30s-STS (n) | 20.1 ± 4.8 |

SD, standard deviation; BMI, body mass index; ASMI, appendicular skeletal muscle index; SPPB, Short Physical Performance Battery; 30-STS, 30-second sit-to-stand test; KES, knee extensor strength; HGS, handgrip strength; TUG, timed up and go test

**Table S3.** Agreement of pose-estimated peak power and joint angles with the reference method (participant-level means).

| **Variables** | **Pose-estimation** | **Reference** | **P-value** | **ICC (2,1) (95% CI)** |
| --- | --- | --- | --- | --- |
| Peak power | 510.7 ± 142.7 | 472.7 ± 133.9 | <0.001 | 0.94 (0.85 to 0.96) |
| KF_max_ | 83.1 ± 6.0 | 85.3 ± 6.2 | 0.003 | 0.84 (0.64 to 0.93) |
| KF_min_ | 9.4 ± 5.4 | 11.7 ± 5.4 | <0.001 | 0.83 (0.60 to 0.91) |
| TF_max_ | 27.5 ± 7.4 | 24.4 ± 6.1 | <0.001 | 0.83 (0.64 to 0.91) |
| TF_min_ | −0.2 ± 6.1 | −2.6 ± 6.2° | <0.001 | 0.89 (0.74 to 0.94) |

Values are grand means ± between-subject SD. P-values are from paired t-tests between pose-estimation and the reference method. ICC, intraclass correlation coefficient; CI, confidence interval; SD, standard deviation.

**Table S4.** Differences in sit-to-stand metrics according to sarcopenia status and comorbidities

| **Category** | **N** | **Peak P_PE_** | **5x-STS** | **30s-STS** |
| --- | --- | --- | --- | --- |
| Sarcopenia status: AWGS 2019 criteria |  |  |  |  |
| Sarcopenia + severe sarcopenia | 10 | -37.8 ± 27.3 | 1.27 ± 0.84 | -2.9 ± 1.5 |
| Probable sarcopenia | 50 | -44.1 ± 14.9* | 0.58 ± 0.46 | -1.7 ± 0.8* |
| Non-sarcopenia | 69 | (Reference) |  |  |
| Sarcopenia status: EWGSOP2 criteria |  |  |  |  |
| Sarcopenia + severe sarcopenia | 5 | -57.2 ± 38.0 | 2.41 ± 1.14 | -3.6 ± 2.0 |
| Probable sarcopenia | 28 | -10.0 ± 17.8 | -0.29 ± 0.53 | -0.7 ± 1.0 |
| Non-sarcopenia | 96 | (Reference) |  |  |
| Number of comorbidities |  |  |  |  |
| 2 or more | 57 | 10.9 ± 18.4 | 0.07 ± 0.55 | -0.5 ± 1.0 |
| 1 | 35 | 6.7 ± 19.7 | 0.64 ± 0.59 | -0.2 ± 1.1 |
| 0 | 37 | (Reference) |  |  |
| Hypertension |  |  |  |  |
| Yes | 58 | 26.7 ± 15.5 | -0.25 ± 0.47 | 0.1 ± 0.8 |
| No | 71 | (Reference) |  |  |
| Dyslipidemia |  |  |  |  |
| Yes | 58 | -24.1 ± 15.1 | 0.55 ± 0.46 | -1.2 ± 0.8 |
| No | 71 | (Reference) |  |  |
| Diabetes |  |  |  |  |
| Yes | 25 | 11.7 ± 18.6 | -0.13 ± 0.56 | -0.3 ± 1.0 |
| No | 104 | (Reference) |  |  |
| Heart disease |  |  |  |  |
| Yes | 12 | -9.0 ± 25.3 | 0.84 ± 0.76 | 0.0 ± 1.4 |
| No | 117 | (Reference) |  |  |
| Previous history of cancer |  |  |  |  |
| Yes | 12 | 41.2 ± 25.3 | -0.65 ± 0.77 | 0.4 ± 1.4 |
| No | 117 | (Reference) |  |  |
| Any of Thyroid / Liver / Lung / Kidney disease |  |  |  |  |
| Yes | 13 | -12.5 ± 24.6 | -0.12 ± 0.74 | -0.2 ± 1.3 |
| No | 116 | (Reference) |  |  |

Peak P_PE_, 5x-STS, and 30s-STS were compared across categories of sarcopenia (AWGS 2019 and EWGSOP2 criteria) and major comorbidities using analysis of covariance with sex and body mass as covariates. Values are adjusted mean differences (β ± SE) relative to the reference group. Peak P_PE_, pose-estimation–derived peak STS power; 5x-STS, five-times sit-to-stand test; 30s-STS, 30-second sit-to-stand test; AWGS 2019, Asian Working Group for Sarcopenia 2019; EWGSOP2, European Working Group on Sarcopenia in Older People 2.

*P < 0.05 versus the reference group.

**Table S5.** Pearson correlations between sit-to-stand metrics and clinical measures among participants with probable sarcopenia, sarcopenia, or severe sarcopenia (AWGS 2019 criteria)

|  | Peak P_PE_ |  | 5x-STS |  | 30s-STS |  |
| --- | --- | --- | --- | --- | --- | --- |
|  | r | P-value | r | P-value | r | P-value |
| Physical performance |  |  |  |  |  |  |
| Usual walking speed | 0.14 | 0.287 | -0.34 | 0.008 | 0.29 | 0.023 |
| TUG velocity | -0.06 | 0.643 | -0.62 | <0.001 | 0.46 | <0.001 |
| SPPB | 0.11 | 0.395 | -0.79 | <0.001 | 0.56 | <0.001 |
| Muscle strength |  |  |  |  |  |  |
| Knee extensor strength | 0.67 | <0.001 | -0.21 | 0.102 | 0.35 | 0.006 |
| Handgrip strength | 0.65 | <0.001 | -0.23 | 0.330 | 0.21 | 0.101 |
| Muscle mass |  |  |  |  |  |  |
| ASMI | 0.76 | <0.001 | 0.01 | 0.950 | 0.22 | 0.088 |

5x-STS, five-times sit-to-stand test; 30-s STS, 30-second sit-to-stand test; TUG, Timed Up and Go; ASMI, appendicular skeletal muscle index; SPPB, Short Physical Performance Battery; Peak P_PE_, pose-estimation–derived peak STS power.
