# Supplementary material for: Sit‐to‐Stand Power From 2D Pose Estimation as an Indicator of Muscle Strength in Older Adults
Source: J Cachexia Sarcopenia Muscle. 2026 Jan 26;17(1):e70208. doi: 10.1002/jcsm.70208 (PMC12834695; doi:10.1002/jcsm.70208)
Supplement: Supplementary file 2 — Figure S1: Ensemble trajectories for (a) STS power, (b) knee flexion angle and (c) trunk flexion angle. Blue solid line: pose estimation; red solid line: reference; grey dashed line: pointwise RMSE over the normalized cycle; curves: grand mean across participants; grey shaded band: grand mean ± SDbetween. Figure S2: Validation of peak joint angles from pose estimation. Participant‐level mean (a) KFmax, (b) KFmin, (c) TFmax and (d) TFmin from pose estimation plotted against motion capture. Black solid line: fitted regression; grey shaded band: 95% confidence interval. Figure S3: Bland–Altman plots for participant‐level means of (a) KFmax, (b) KFmin, (c) TFmax and (d) TFmin. The black dashed line marks the mean bias, and the grey dotted lines indicate the upper and lower 95% limits of agreement. Figure S4: Linear mixed‐effects model for male. Scatterplots of within‐subject associations between ΔPeak PPE (%) and (a) ΔKFmax, (b) ΔKFmin, (c) ΔTFmax, (d) ΔTFmin and (e) Tpeak. Black solid line: fixed‐effect fit; grey shaded band: 95% CI for fixed effect. Figure S5: Linear mixed‐effects model for female. Scatterplots of within‐subject associations between ΔPeak PPE (%) and (a) ΔKFmax, (b) ΔKFmin, (c) ΔTFmax, (d) ΔTFmin and (e) Tpeak. Black solid line: fixed‐effect fit; grey shaded band: 95% CI for fixed effect. [file JCSM-17-e70208-s001.docx]

**
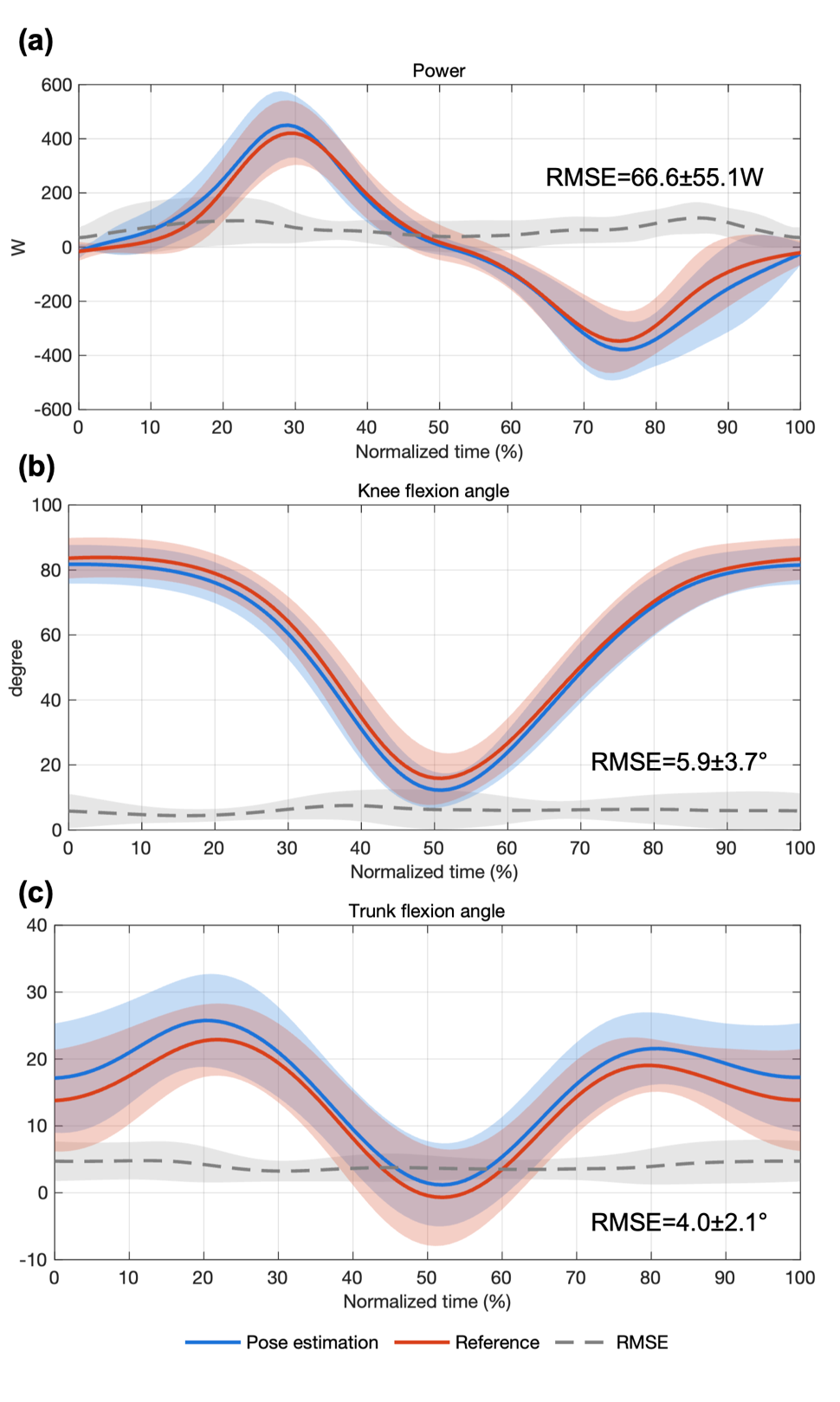
**

**Figure S1.** Ensemble trajectories for (a) STS power, (b) knee flexion angle, and (c) trunk flexion angle. Blue solid line: pose estimation; red solid line: reference; gray dashed line: pointwise RMSE over the normalized cycle; Curves: grand mean across participants; gray shaded band: grand mean ± SD_between_.


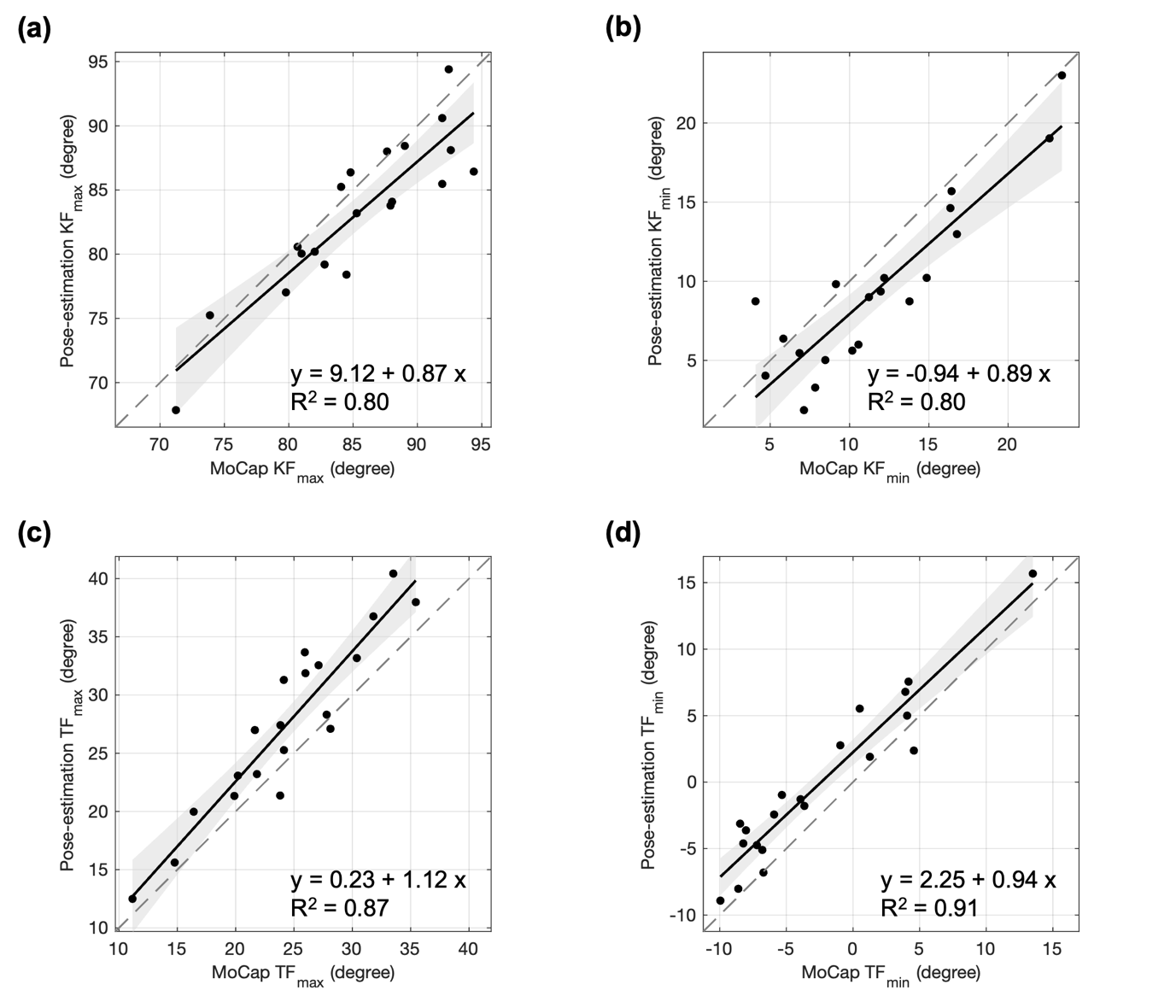


**Figure S2.** Validation of peak joint angles from pose estimation. Participant-level mean (a) KFmax, (b) KFmin, (c) TFmax, and (d) TFmin from pose estimation plotted against motion capture. Black solid line: fitted regression; gray shaded band: 95% confidence interval.


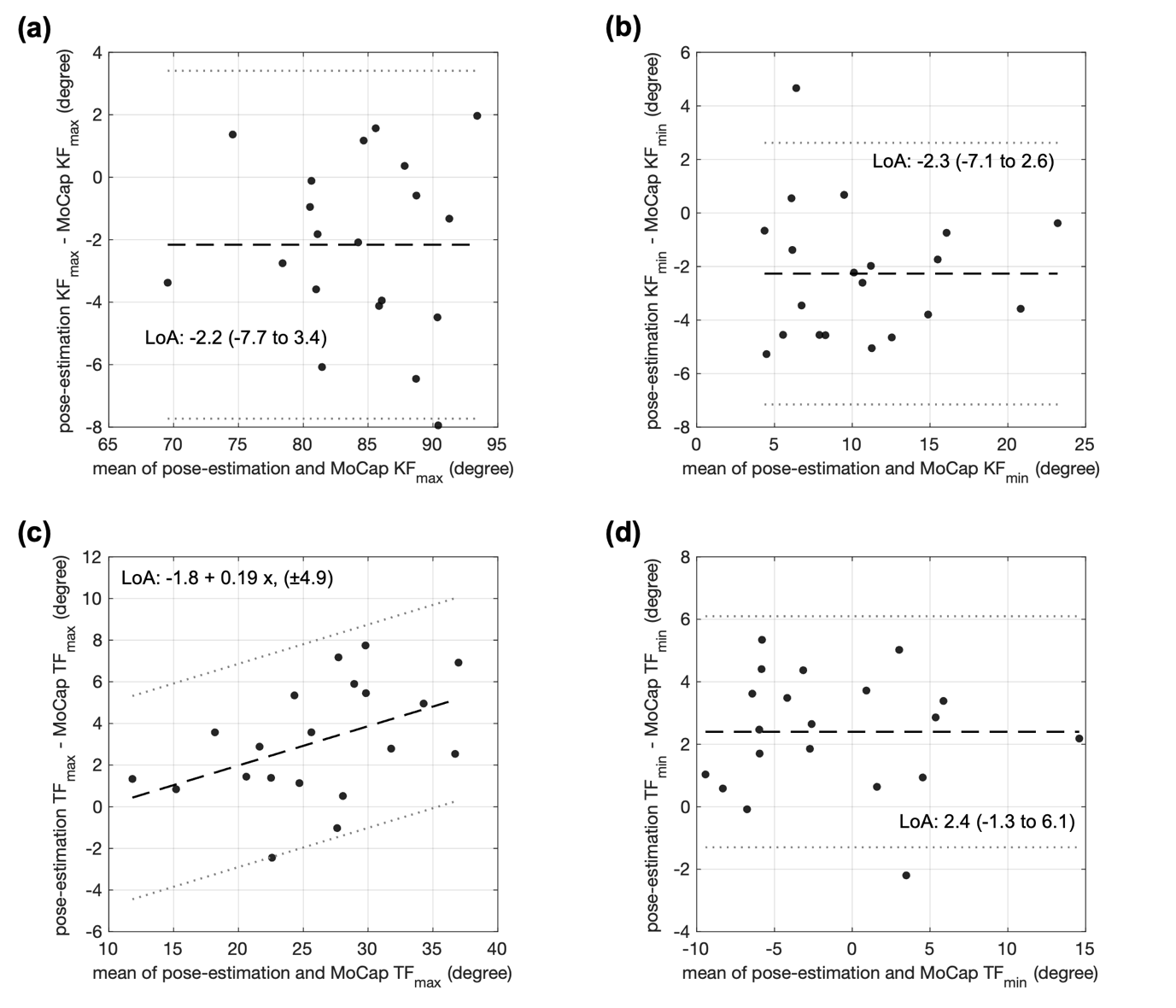


**Figure S3.** Bland–Altman plots for participant-level means of (a) KF_max_, (b) KF_min_, (c) TF_max_, and (d) TF_min_. The black dashed line marks the mean bias, and the gray dotted lines indicate the upper and lower 95% limits of agreement.


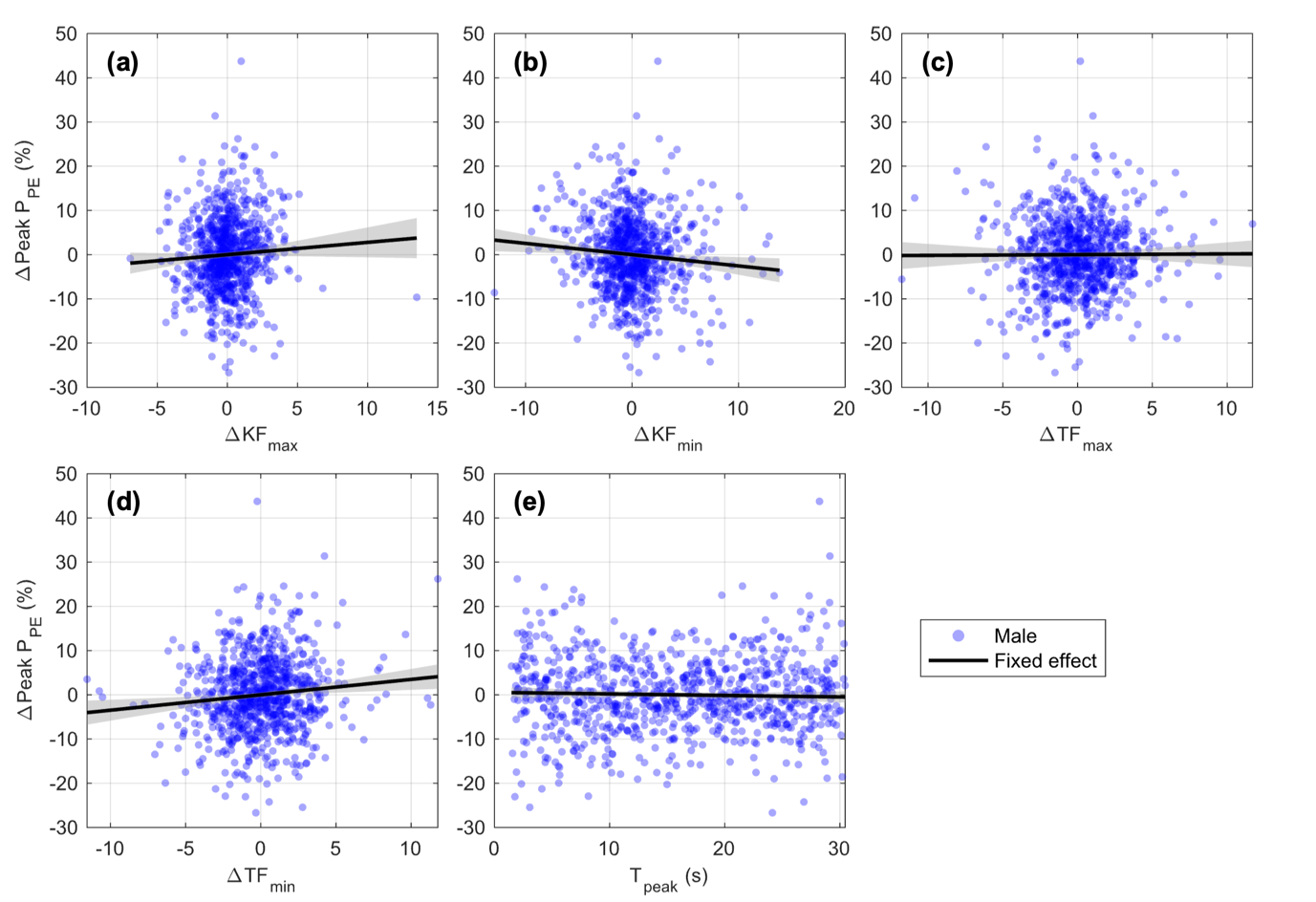


**Figure S4.** Linear mixed-effects model for male. Scatterplots of within-subject associations between ΔPeak P_PE_ (%) and (a) ΔKF_max_, (b) ΔKF_min_, (c) ΔTF_max_, (d) ΔTF_min_, and (e) T_peak_. Black solid line: fixed-effect fit; Gray shaded band: 95% CI for fixed effect.


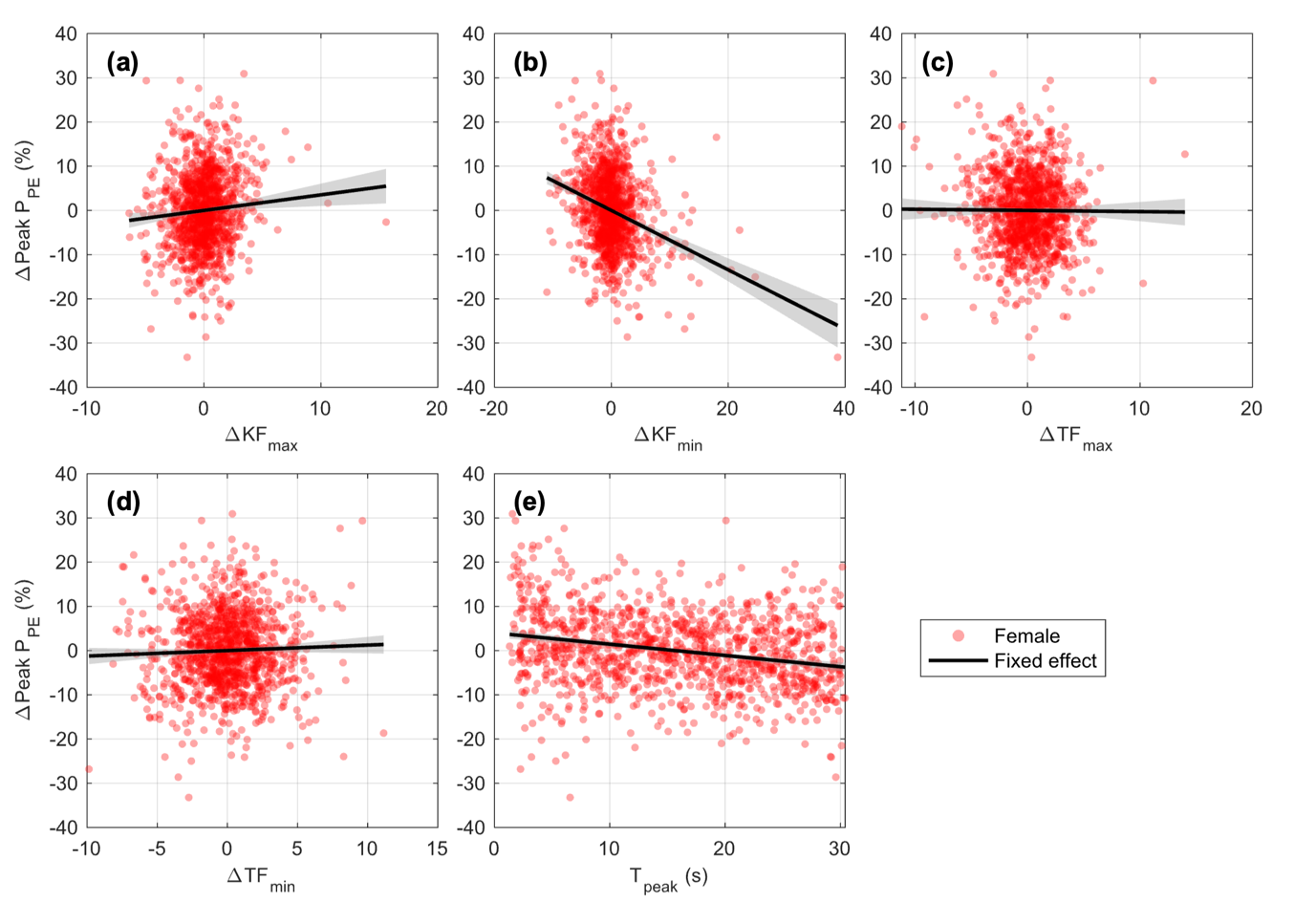


**Figure S5.** Linear mixed-effects model for female. Scatterplots of within-subject associations between ΔPeak P_PE_ (%) and (a) ΔKF_max_, (b) ΔKF_min_, (c) ΔTF_max_, (d) ΔTF_min_, and (e) T_peak_. Black solid line: fixed-effect fit; Gray shaded band: 95% CI for fixed effect.
